# Development of core outcome sets for effectiveness trials focused on infants and children with wasting and nutritional oedema

**DOI:** 10.1136/bmjgh-2024-017225

**Published:** 2025-10-02

**Authors:** Allison I Daniel, Kirrily de Polnay, Michael McCaul, Jaden Bendabenda, Zita Weise Prinzo, Celeste E Naude

**Affiliations:** 1 Consultant to the World Health Organization, Geneva, Switzerland; 2 Department of Nutrition and Food Safety, World Health Organization, Geneva, Switzerland; 3 Centre for Evidence-based Health Care, Division Epidemiology and Biostatistics, Department of Global Health, Stellenbosch University, Cape Town, South Africa

**Keywords:** Child health, Nutrition, Intervention study, Systematic review, Global Health

## Abstract

**Introduction:**

The 2023 WHO guideline on the prevention and management of wasting and nutritional oedema includes recommendations informed by best evidence from systematic reviews addressing critical and important outcomes. Multiple outcomes across trials were a challenge during guideline development, highlighting a need to establish core outcome sets (COS) for wasting and nutritional oedema. Informed by Core Outcome Measures in Effectiveness Trials (COMET) Initiative best practice methods, we aimed to develop six COS for effectiveness trials around prevention and management of wasting and nutritional oedema in infants and children.

**Methods:**

Guideline Development Group and UNICEF/WHO Technical Advisory Group on Wasting and Nutritional Oedema members were invited to participate in a Delphi process to establish these COS. This involved scoring outcomes in two rounds of a survey (Likert scale from 1 to 9), with consensus in the second round defined as a minimum of 70% of the expert panel giving a score of at least 7 and <15% scoring 3 or below.

**Results:**

Twenty-five of 36 invited participants completed each survey, followed by multiple consensus meetings to reach agreement on the outcomes. Through this Delphi process, we developed six COS for infants <6 months of age with wasting and/or nutritional oedema and/or underweight in (1) inpatient and (2) outpatient/community settings, infants and children 6–59 months of age with severe wasting and/or nutritional oedema in (3) inpatient and (4) outpatient/community settings, infants and children 6–59 months of age with moderate wasting in (5) outpatient/community settings and (6) prevention of wasting and nutritional oedema.

**Conclusion:**

Primary research, future guidelines and related decision-making stand to be strengthened by these six COS, including the most critical outcomes from a child health perspective to be evaluated in future effectiveness trials on wasting and nutritional oedema. Uptake of these COS will inform their further development.

**WHAT IS ALREADY KNOWN ON THIS TOPIC**

- There is a lack of standardisation of outcome measurement and reporting for effectiveness trials on prevention and management of wasting and nutritional oedema, which made for challenges during the guideline development process for the 2023 WHO guideline on wasting and nutritional oedema in infants and children.
- The Core Outcome Measures in Effectiveness Trials (COMET) Initiative defines a core outcome set (COS) as an ‘agreed standardised set of outcomes that should be measured and reported, as a minimum, in all clinical trials in specific areas of health or healthcare’.

**WHAT THIS STUDY ADDS**

- Based on COMET Initiative best practice methods, we used a rigorous, transparent Delphi process to develop six COS for effectiveness trials on wasting and nutritional oedema, which include the most critical outcomes from a child health perspective, along with guidance and key points that emerged from multiple consensus meetings.

**HOW THIS STUDY MIGHT AFFECT RESEARCH, PRACTICE OR POLICY**

- These COS are intended to be used by researchers to ensure that at a minimum, data for these critical outcomes are reported for all effectiveness trials in infants and children with wasting and nutritional oedema.
- Uptake of these COS will strengthen primary research, future guidelines and related decision-making, including informing recommendations on wasting and nutritional oedema as updates to the 2023 WHO guideline.
- This is the first time that COS have been developed for infants and children with any form of malnutrition and feedback from those applying the COS will inform further understanding, utility and future development.

## Introduction

 The *WHO guideline on the prevention and management of wasting and nutritional oedema (acute malnutrition) in infants and children under 5 years,*^1^ published in 2023, was developed in line with WHO’s standards and methods for guideline development and following the Grading of Recommendations, Assessment, Development and Evaluations (GRADE) approach for determining the certainty of evidence and for formulating recommendations based on this evidence.^1 2^ The scope of the guideline includes the following four areas of focus:

- Management of infants <6 months of age at risk of poor growth and development.
- Management of moderate wasting in infants and children 6–59 months of age.
- Management of severe wasting and/or nutritional oedema in infants and children 6–59 months of age.
- Prevention of wasting and nutritional oedema.

Scoping reviews for the four areas were completed and, using a prioritisation approach, 16 guideline questions across the above four areas were prioritised by a Guideline Development Group (GDG) of 27 experts, which is further described in the 2023 WHO guideline and separate papers that include information about the guideline development process.^1 3 4^ This was followed by prioritisation of critical and important outcomes for each guideline question by the GDG, in line with GRADE guidance.^5 6^ Specifically, GDG members were invited to score a list of outcomes for the guideline questions using a Likert scale from 1 to 9, with scores from 1 to 3 indicating ‘not important’, scores from 4 to 6 indicating ‘important but not critical’, and scores from 7 to 9 indicating ‘critical importance’. Up to seven outcomes/outcome domains were prioritised (only critical and important) for each respective guideline question. One of the six guiding principles established for the guideline development process emphasised the importance of a child health perspective (children’s health, growth and development at the forefront), and this perspective was taken when prioritising outcomes. After outcome prioritisation, systematic reviews were commissioned for guideline question to identify best available evidence for the prespecified outcomes.

There were several challenges encountered by the systematic reviewers and the GDG at the stage of making judgements through the Evidence-to-Decision framework step of the guideline development process.^6^ First, many outcomes reported by trials were not well defined, with large variations in definitions, criteria and measurements being used across trials, such as for the outcome relapse.^7^ Consequently, pooling and interpreting the body of evidence around effects on a specific prespecified outcome were difficult.

Anthropometry was prioritised for most guideline questions. Managing an outcome domain with numerous different outcome measures being reported across trials was challenging. These included weight-for-length z-scores or weight-for-height z-scores (WLZ or WHZ), mid-upper arm circumference (MUAC), MUAC change (eg, millimetre per day or week), weight, rate of weight change (eg, grams per kilogram per day), weight-for-age z-scores (WAZ), length-for-age z-scores or height-for-age z-scores and more. The range of outcome measures used by trials to report effects on anthropometry meant that evidence of effects on anthropometry was often thinly spread across many outcomes. In some cases, the GDG had to make judgements on the evidence across 12 or more outcomes, many of which had some collinearity and represented similar constructs but could not be pooled.

Furthermore, there is no clear agreement about the importance of anthropometric outcomes and their predictive validity or prognostic value in infants and children with wasting and/or nutritional oedema. There are also questions about the value of anthropometric outcomes relative to other outcomes of interest.^8 9^ These could include longer term outcomes like sustained recovery, which may be more challenging to measure but could be advocated for if there is agreement through a structured and transparent consensus process.

Our aim was, therefore, to develop core outcome sets (COS) related to the areas of focus in the 2023 WHO guideline on wasting and nutritional oedema through a Delphi process. The Core Outcome Measures in Effectiveness Trials (COMET) Initiative defines a COS as an ‘agreed standardised set of outcomes that should be measured and reported, as a minimum, in all clinical trials in specific areas of health or healthcare’. Importantly, using a COS does not imply that outcomes in a study should be restricted to those in the relevant COS. Rather, there is an expectation that data on the core outcomes will be collected and reported.^10^

## Methods

The COS development was led by the 2023 WHO guideline Steering Committee and methodologists for the WHO guideline on wasting and nutritional oedema, following COMET best practice methods including eliciting views about critical outcomes in a consensus process with an expert panel.^10^ We registered the project *a priori* in the COMET Initiative database (https://www.comet-initiative.org/Studies/Details/3121) and reported results according to the Core Outcome Set Standards for Reporting to ensure transparent and complete reporting of the COS development (online supplemental Material).^11^

### COS for wasting and nutritional oedema

We aimed to develop six-related COS for effectiveness trials on each of the below populations and settings for management of wasting and nutritional oedema (COS 1–5) and prevention of wasting and nutritional oedema (COS 6):

1. Infants <6 months of age with wasting (WLZ below −2 SD) and/or nutritional oedema and/or underweight (WAZ below −2 SD)—*interventions in inpatient settings*.
2. Infants <6 months of age with wasting (WLZ below −2 SD) and/or nutritional oedema and/or underweight (WAZ below −2 SD)—*interventions in outpatient/community settings.*
3. Infants and children 6–59 months of age with severe wasting (WHZ or WLZ below −3 SD and/or MUAC <115 mm) and/or nutritional oedema—*interventions in inpatient settings*.
4. Infants and children 6–59 months of age with severe wasting (WHZ or WLZ below −3 SD and/or MUAC <115 mm) or nutritional oedema—*interventions in outpatient/community settings.*
5. Infants and children 6–59 months of age with moderate wasting (WHZ or WLZ between −3 SD and below −2 SD and/or MUAC between 115 mm and <125 mm)—*interventions in outpatient/community settings*.
6. Prevention of wasting (WLZ below −2 SD and/or MUAC <125 mm) and nutritional oedema.

Infants <6 months of age at risk of poor growth and development were included in the 2023 WHO guideline as a key population of interest, and there are significant evidence gaps for this group of infants with research being urgently required. However, it would be challenging to establish COS appropriate and relevant for all infants meeting any of the multiple criteria for poor growth and development.^1^ Therefore, the focus of the COS 1 and 2 was on infants <6 months of age with wasting and/or nutritional oedema and/or underweight.

### Participants in the expert panel for the Delphi process

We invited members of the GDG for the 2023 WHO guideline and the UNICEF/WHO Technical Advisory Group (TAG) on Wasting and Nutritional Oedema to participate in the COS development process.^12^ Each of these people has completed standard declaration of interest forms in alignment with WHO’s guidelines for declarations of interests for WHO experts.^13^ These declaration of interest statements were reviewed by the responsible WHO technical officers. Participants were also asked to declare any conflicts of interest at the start of each consensus meeting.

The GDG included 27 multidisciplinary experts^1^ and the TAG includes 18 multidisciplinary experts.^12^ Some experts are members of both groups, bringing the total number of potential participants to 36. These experts are based across all six WHO regions and have research, clinical, programmatic and policy experience related to wasting and nutritional oedema in diverse settings. Many participants are directly involved in clinical care of infants and children with wasting and/or nutritional oedema. Participants did not necessarily have to be researchers to be able to contribute to this process. They were considered a single panel, and no further data were collected about the characteristics of the expert panel.

Prior to starting with COS development, we shared resources about the Delphi process being undertaken to establish the COS and held a meeting with potential participants, including a dedicated question-and-answer period. This meeting was recorded and shared with all potential participants.

This COS process did not require submission to the WHO Research Ethics Review Committee as confirmed by a committee representative, nor did it require informed consent as it did not meet WHO’s criteria for research with human participants.

### Patient and public involvement

Patients and caregivers were not involved in this process to develop COS.

### Collation of initial long lists of outcomes for the COS

Initial outcome long lists were generated for each COS starting with all outcomes prioritised for 13 effectiveness guideline questions for the 2023 WHO guideline, regardless of whether evidence was available for the outcome during guideline development. Specific outcomes within relevant broader domains that were identified in the 13 effectiveness systematic reviews commissioned for these guideline questions were also added. We carried over outcomes from one COS population to another if we considered these to be applicable to more than one population of interest, with an example being child development.

Protocols for the systematic reviews commissioned for the WHO guideline were published in PROSPERO (the International Prospective Register of Systematic Reviews) after input from members of the WHO guideline Steering Committee and methodologists to ensure relevance and applicability to the guideline questions and process. Members of the WHO Steering Committee and methodologists also gave regular guidance around the systematic reviews. GRADE Evidence Profiles were reviewed by the GDG during the guideline development process.^6 14^ Systematic reviews for the guideline were completed between 2021 and mid-2023.^15^

To facilitate and provide sensible structure to the surveys and decision-making for the COS development process, we categorised the collated long lists of specific outcomes into outcome domains. These outcome domains were agreed on by WHO Steering Committee and methodologists. These domains included mortality, anthropometric outcomes, breastfeeding outcomes, morbidity, hospital stay, adverse events, clinical outcomes, care pathway outcomes, child development and incidence and prevalence (for COS 6 on prevention). Some of these domains followed the COMET outcome taxonomy. However, there were many outcomes related to wasting and nutritional oedema for which the taxonomy was not applicable and we were, therefore, unable to categorise many outcomes within this taxonomy.^16^

### Approach to COS development through a Delphi process

The virtual Delphi process we used to develop the six COS consisted of two rounds of a survey followed by multiple consensus meetings.

### Online survey with two rounds

We used web-based software QuestionPro (https://www.questionpro.com/) to implement the survey. At the start of each survey round, potential participants were sent an individual invitation email with the protocol and the relevant survey link. We included specific instructions within each survey round, including what differed between the two different surveys. Participants in the expert panel were given 3 weeks to complete each round with at least two reminder emails sent before the deadline for each round. They were invited to respond to round 2 even if they did not participate in round 1. Names of participants were kept confidential throughout.

Round 1 involved scoring all outcomes in the initial long lists, which were categorised by outcome domains described earlier for each of the COS. The expert panel scored outcomes based on a Likert scale from 1 to 9. As was the case for outcome rating in the guideline development process, scores from 1 to 3 meant ‘not important’, scores from 4 to 6 meant ‘important but not critical’, and scores from 7 to 9 meant ‘critical importance’.^5 6^ Participants were able to select ‘don’t know’ if they felt unable to score any specific outcomes.

The expert panel was made aware in the instructions that the long lists of outcomes for the COS were based on the questions prioritised and systematic reviews commissioned for the guideline and may not be exhaustive. For each COS, an open text box was provided for additional outcomes that participants could optionally suggest being included in the next round. We added all outcomes suggested during the first round of the survey to the second round.

For round 2, participants were sent their own results from round 1 and the overall results from the whole expert panel. The overall results were shared as means (SD) and medians (IQR). Participants in the expert panel were invited in this round to rescore all outcomes based on the same Likert scale. They were asked to also score additional outcomes suggested by participants in the first round. There was a text box for each COS where participants could add questions or comments and give reasons for changing any scores between the first and second rounds. Additional outcomes could not be suggested in round 2.

In both survey rounds, a text box for each COS was provided for participants to add questions or comments, such as rationale for giving a certain score to specific outcomes. These questions and comments were recorded by the WHO Steering Committee and methodologists for discussion at the consensus meetings if they were relevant to the outcomes that met the criteria for consensus.

We examined the results of round 2 to determine if there was consensus for the outcomes for each COS. Consensus for inclusion of an outcome was defined as a minimum of 70% of participants in the expert panel giving a score of at least 7 and <15% of participants scoring 3 or below.^10 17^

### Consensus meetings

After completion of the online survey, we scheduled multiple consensus meetings with the expert panel to finalise the six COS, which were hosted and recorded using Zoom Workplace (V.6.2.5). Outcomes that met consensus from the online survey for each COS were shared in advance with the expert panel and again during the meetings. The first objective of the consensus meetings was to further narrow down the outcomes for each of the six COS, aiming for a maximum of seven outcomes; the second objective was to agree on definitions, measures and measurement of the final agreed outcomes included in the 6 COS. In the interest of time, we were unable to discuss outcomes that did not meet criteria for consensus from the surveys.

For the first consensus meeting objective, we went through each COS separately using the same approach. The expert panel was presented with the list of outcomes that met consensus following the online survey and asked to first vote on each outcome using a 3-point Likert scale using anonymous Zoom polling, with options being ‘not important’, ‘important but not critical’ and ‘critical importance’. This approach has been shown to be effective in reducing the number of outcomes identified as critical and allowing for narrowing down the list.^18^ If at least 70% of participants scored an outcome as critical during this poll, the outcome would move forward for possible inclusion in the COS. After this poll, regardless of the result, the expert panel was invited to share any arguments in favour of or against inclusion of the outcome in the COS, or any other important details related to the outcome. After discussion, if deemed necessary based on whether there was discordance or ambiguity in the initial vote, participants were invited to vote again using the 3-point Likert scale, with additional discussion following. If required, additional surveys using the same 3-point Likert scale were used.

On completion of this process for each respective COS, we allowed time for discussion about the entirety of the list of final outcomes to ensure there was agreement. Once all COS were completed and considering the related nature of the COS, we displayed the final six COS alongside each other for reflection and further discussion and shared these with the expert panel following the meeting for final review.

We also gave opportunity for input from the expert panel to agree on definitions/specific measures and measurement of outcomes that are included in the 6 COS. We documented and consolidated guidance and key points about outcome definitions, measurement and reporting raised by participants throughout the consensus meetings, drawing on the recordings of the meetings. Participants also had the opportunity to review the consolidated guidance and key points after all the meetings were concluded.

If outcomes involved using a measurement tool, we anticipated allowing for discussion of good measurement properties if applicable to the outcomes in the COS^19^; but this was not needed for any outcomes that were included in the final COS since there were no specific outcomes that require the use of questionnaires, scales, performance-based tests or biomarkers, for example.

## Results

### Participants in an expert panel for the COS development process

25 out of 36 invited participants completed each the round 1 survey between June and July 2024 and the round 2 survey between July and August 2024. These respondents were based in 21 countries, including 14 low and middle-income countries. The eight consensus meetings between October and November 2024 had participation of 24 people from 17 countries including 13 low and middle-income countries, with an average of 13 participants per meeting.

### COS developed through the Delphi process

There were 43 unique outcomes from the systematic reviews for the 13 effectiveness guideline questions. New outcomes suggested by participants in round 1 of the survey and added to round 2 of the survey included body composition, oedema resolving, loss to follow-up or default, successful transfer to outpatient care, other functional outcomes, immune function biomarkers, gut inflammation biomarkers, maternal mental health and appetite. The complete list of outcomes included in the surveys with the survey results is included in online supplemental material.

We also assessed whether there was a reduction in variability in scores between the first and second rounds, which was the case. The number of outcomes that met criteria for consensus after round 2 of the survey and after being further narrowed down in the consensus meetings to the final outcomes is shown in online supplemental table 1 for each COS. Outcomes that were excluded during the consensus meetings are provided in online supplemental material.

Table 1 provides an overview of the five COS for effectiveness trials focused on management of infants and children with wasting and nutritional oedema (COS 1–5). Some outcomes were deemed critical for inclusion in all COS on management including mortality and sustained recovery, while others were more relevant for particular populations and settings along the continuum of care, such as oedema resolving and breastfeeding indicators, as examples.

**Table 1:** Overview of core outcome sets for effectiveness trials focused on management of infants and children with wasting and nutritional oedema

1) Infants <6 months of age with wasting and/or nutritional oedema and/or underweight***—**inpatient settings*	2) Infants <6 months of age with wasting and/or nutritional oedema and/or underweight***—**outpatient/community settings*	3) Infants and children 6–59 months of age with severe wasting and/or nutritional oedema*—inpatient settings*	4) Infants and children 6–59 months of age with severe wasting or nutritional oedema*—outpatient/community settings*	5) Infants and children 6–59 months of age with moderate wasting*—outpatient/community settings*
Mortality	Mortality	Mortality	Mortality	Mortality
Weight change (g/kg/day)	Weight change (g/kg/day)		Weight change (g/kg/day)	Weight change (g/kg/day)
	Mid-upper arm circumference (MUAC)		MUAC	MUAC
	Weight-for-age z-scores (WAZ)		WAZ	WAZ
Oedema resolving		Oedema resolving	Oedema resolving	
Clinical improvement		Clinical improvement		
Clinical deterioration		Clinical deterioration		
Recovery from morbidity	Morbidity or recovery from morbidity	Recovery from morbidity		
Breastfeeding indicators	Breastfeeding indicators			
	Anthropometric recovery*		Anthropometric recovery	Anthropometric recovery
			Time to anthropometric recovery	
	Non-response*		Non-response	Non-response
				Deterioration to severe wasting and/or nutritional oedema
Sustained recovery	Sustained recovery*	Sustained recovery	Sustained recovery	Sustained recovery

* Applies to infants with wasting and/or nutritional oedema, rather than infants with underweight only.

The consolidated guidance and key points about outcome definitions, measurement and reporting raised by the expert panel during the consensus meetings were tabulated. The expert panel had continuous discussions during the consensus meetings about how prescriptive or rigid the definitions, measurement and reporting should be and agreed on flexibility, with the rationale clearly documented. Tables2^6^ present the outcomes for each of these respective COS along with guidance and key points about outcome measurement and reporting. Table 7 includes the COS for prevention of wasting and nutritional oedema (COS 6) with guidance and key points about outcome measurement and reporting. Additional discussion points from the consensus meetings about outcome measurement and reporting are presented narratively by outcome domain.

**Table 2:** Core outcome set (1) Infants <6 months of age with wasting and/or nutritional oedema and/or underweight—*interventions in inpatient settings*

Outcomes	Guidance and key points about outcome measurement and reporting*
Mortality	All-cause mortality, to be reported as a binary outcome
Weight change (g/kg/day)	Rate of weight change in grams per kilogram body weight per day (g/kg/day), to be reported as a continuous outcome While weight change is considered a critical outcome for this population, the optimal rate of weight gain has not yet been established
Oedema resolving	Bilateral oedema grade improved or resolution of bilateral oedema, to be reported as a binary outcome
Clinical improvement	Based on clinical and nutritional signs, to be reported as a binary outcome
Clinical deterioration	Based on clinical signs or need for clinical interventions/support, to be reported as a binary outcome Reporting of clinical deterioration is an ethical requirement for trials, and is also seen as a critical outcome
Recovery from morbidity	Resolution of medical problems that prompted admission for inpatient care, to be reported as a binary outcome
Breastfeeding indicators	For example, exclusive breastfeeding in infants <6 months of age, to be reported as a binary outcome
Sustained recovery	Anthropometric recovery sustained for at least 6 months ideally, to be reported as a binary outcome This outcome should be reported in any trial with post-exit anthropometric outcomes for as long as infants were followed up after exit If the follow-up time is shorter or data are unable to be reported, researchers should include a statement in the limitations section Reporting on sustained recovery also accounts for relapse and readmission, which were not individually deemed critical outcomes

* Guidance and key points about outcome measurement and reporting emerged from discussion with the expert panel during the consensus meetings.

**Table 3:** Core outcome set (2) Infants <6 months of age with wasting and/or nutritional oedema and/or underweight—*interventions in outpatient/community settings*

Outcomes	Guidance and key points about outcome measurement and reporting*
Mortality	All-cause mortality, to be reported as a binary outcome
Weight change (g/kg/day)	Rate of weight change in grams per kilogram body weight per day (g/kg/day), to be reported as a continuous outcome While weight change is considered a critical outcome for this population, the optimal rate of weight gain has not yet been established
Mid-upper arm circumference (MUAC)	MUAC in mm, to be reported as a continuous outcome
Weight-for-age z-scores (WAZ)	WAZ based on the 2006 WHO child growth standards, to be reported as a continuous outcome
Morbidity or recovery from morbidity	Episodes of morbidity or resolution of medical problems, to be reported as a continuous outcome if capturing the number of episodes or as a binary outcome for resolution of medical problems
Breastfeeding indicators	For example, exclusive breastfeeding in infants <6 months of age, to be reported as a binary outcome
Anthropometric recovery†	Recovery based on anthropometric indicators, to be reported as a binary outcome
Non-response†	Not achieving anthropometric recovery within 4 months of initiating treatment, to be reported as a binary outcome
Sustained recovery†	Anthropometric recovery sustained for at least 6 months ideally, to be reported as a binary outcome This outcome should be reported in any trial with post-exit anthropometric outcomes for as long as infants were followed up after exit; the duration of follow-up may be dependent on the type of intervention (eg, dietary or non-dietary) If the follow-up time is shorter or data are unable to be reported, researchers should include a statement in the limitations section Reporting on sustained recovery also accounts for relapse and readmission, which were not individually deemed critical outcomes

* Guidance and key points about outcome measurement and reporting emerged from discussion with the expert panel during the consensus meetings.

† Applies to infants with wasting and/or nutritional oedema, rather than infants with underweight only.

**Table 4:** Core outcome set (3) Infants and children 6–59 months of age with severe wasting and/or nutritional oedema—*interventions in inpatient settings*

Outcomes	Guidance and key points about outcome measurement and reporting*
Mortality	All-cause mortality, to be reported as a binary outcome
Oedema resolving	Bilateral oedema grade improved or resolution of bilateral oedema, to be reported as a binary outcome
Clinical improvement	Based on clinical and nutritional signs, to be reported as a binary outcome
Clinical deterioration	Based on clinical signs or need for clinical interventions/support, to be reported as a binary outcome Reporting of clinical deterioration is an ethical requirement for trials, and is also seen as a critical outcome
Recovery from morbidity	Resolution of medical problems that prompted admission for inpatient care, to be reported as a binary outcome
Sustained recovery	Anthropometric recovery sustained for at least 6 months ideally, to be reported as a binary outcome This outcome should be reported in any trial with post-exit anthropometric outcomes for as long as infants and children were followed up after exit If the follow-up time is shorter or data are unable to be reported, researchers should include a statement in the limitations section Reporting on sustained recovery also accounts for relapse and readmission, which were not individually deemed critical outcomes

* Guidance and key points about outcome measurement and reporting emerged from discussion with the expert panel during the consensus meetings.

**Table 5:** Core outcome set (4) Infants and children 6–59 months of age with severe wasting or nutritional oedema—*interventions in outpatient/community settings*

Outcomes	Guidance and key points about outcome measurement and reporting*
Mortality	All-cause mortality, to be reported as a binary outcome
Weight change (g/kg/day)	Rate of weight change in grams per kilogram body weight per day (g/kg/day), to be reported as a continuous outcome While weight change is considered a critical outcome for this population, the optimal rate of weight gain has not yet been established
Mid-upper arm circumference (MUAC)	MUAC in mm, to be reported as a continuous outcome
Weight-for-age z-scores (WAZ)	WAZ based on the 2006 WHO child growth standards, to be reported as a continuous outcome
Oedema resolving	Bilateral oedema grade improved or resolution of bilateral oedema, to be reported as a binary outcome
Anthropometric recovery	Recovery based on anthropometric indicators, to be reported as a binary outcome
Time to anthropometric recovery	Days until anthropometric recovery has been achieved, to be reported as a continuous outcome
Non-response	Not achieving anthropometric recovery within 4 months of initiating treatment, to be reported as a binary outcome
Sustained recovery	Anthropometric recovery sustained for at least 6 months ideally, to be reported as a binary outcome This outcome should be reported in any trial with post-exit anthropometric outcomes for as long as infants were followed up after exit; the duration of follow-up may be dependent on the type of intervention (eg,dietary or non-dietary) If the follow-up time is shorter or data are unable to be reported, researchers should include a statement in the limitations section Reporting on sustained recovery also accounts for relapse and readmission, which were not individually deemed critical outcomes

* Guidance and key points about outcome measurement and reporting emerged from discussion with the expert panel during the consensus meetings.

**Table 6:** Core outcome set (5) Infants and children 6–59 months of age with moderate wasting—*interventions in outpatient/community settings*

Outcomes	Guidance and key points about outcome measurement and reporting*
Mortality	All-cause mortality, to be reported as a binary outcome
Weight change (g/kg/day)	Rate of weight change in grams per kilogram body weight per day (g/kg/day), to be reported as a continuous outcome While weight change is considered a critical outcome for this population, the optimal rate of weight gain has not yet been established
Mid-upper arm circumference (MUAC)	MUAC in mm, to be reported as a continuous outcome
Weight-for-age z-scores (WAZ)	WAZ based on the 2006 WHO child growth standards, to be reported as a continuous outcome
Anthropometric recovery	Recovery based on anthropometric indicators, to be reported as a binary outcome
Non-response	Not achieving anthropometric recovery within 4 months of initiating treatment, to be reported as a binary outcome
Deterioration to severe wasting and/or nutritional oedema	Progression to severe wasting and/or nutritional oedema based on anthropometric indicators and/or bilateral oedema, to be reported as a binary outcome
Sustained recovery	Anthropometric recovery sustained for at least 6 months ideally, to be reported as a binary outcome This outcome should be reported in any trial with post-exit anthropometric outcomes for as long as infants were followed up after exit; the duration of follow-up may be dependent on the type of intervention (eg,dietary or non-dietary) If the follow-up time is shorter or data are unable to be reported, researchers should include a statement in the limitations section Reporting on sustained recovery also accounts for relapse and readmission, which were not individually deemed critical outcomes

* Guidance and key points about outcome measurement and reporting emerged from discussion with the expert panel during the consensus meetings.

**Table 7:** Core outcome set (6) Prevention of wasting and nutritional oedema

Outcomes	Guidance and key points about outcome measurement and reporting*
Prevalence of wasting and/or nutritional oedema	The proportion of children with wasting (based on MUAC and/or WHZ) and/or nutritional oedema at a specific follow-up time, to be reported as a binary outcome To be included in population- or community-based trials The impact of seasonality must be assessed and should be accounted for if relevant, with prevalence being measured at baseline and follow-up at the same time of year
Prevalence of severe wasting and/or nutritional oedema	The number of children with severe wasting (based on MUAC and/or WHZ) and/or nutritional oedema at a specific follow-up time, to be reported as a binary outcome To be included in population- or community-based trials The impact of seasonality must be assessed and should be accounted for if relevant, with prevalence being measured at baseline and follow-up at the same time of year
Incidence of wasting and/or nutritional oedema	The number of new cases of wasting (based on MUAC and/or WHZ) and/or nutritional oedema over a specific time interval, to be reported as a rate To be included in individually randomised trials, as well as in population-based or community-based trials whenever possible
Incidence of severe wasting and/or nutritional oedema	The number of new cases of severe wasting (based on MUAC and/or WHZ) and/or nutritional oedema over a specific time interval, to be reported as a rate To be included in individually randomised trials, as well as in population- or community-based trials whenever possible
Mid-upper arm circumference (MUAC)	MUAC in mm, to be reported as a continuous outcome
Weight-for-length z-scores (WLZ) or weight-for-height z-scores (WHZ)	WLZ or WHZ based on the 2006 WHO child growth standards, to be reported as a continuous outcome
Morbidity	Presence of morbidity which may be based on inpatient admissions and/or outpatient clinic visits, to be reported as a binary outcome or continuous outcome if capturing multiple visits per child Morbidity data may be of poor quality, and therefore effort should be made to reduce risk of bias around reporting and measurement of this outcome

* Guidance and key points about outcome measurement and reporting emerged from discussion with the expert panel during the consensus meetings.

### Additional points raised during the consensus meetings by outcome domain

#### Mortality in all trials on management of wasting and nutritional oedema

There was agreement from the expert panel to include all-cause mortality in each of the five COS focused on management of wasting and nutritional oedema (COS 1–5). The expert panel recognised that while all trials should report mortality, not all will be powered for mortality as an outcome, nor will all interventions be designed to have an impact on mortality. These points could be explained in trial publications with reference to the respective COS and may be applicable to other outcomes in the COS beyond mortality.

The expert panel also emphasised the importance of reporting loss to follow-up in line with good clinical practice standards, including the 2025 Consolidated Standards of Reporting Trials (CONSORT) guideline, which includes guidance on reporting loss to follow-up,^20^ and for appropriately examining mortality data specifically, even though loss to follow-up was not included as in the COS.

#### Clinical outcomes including deterioration and improvement requiring clinical judgement

Clinical deterioration was identified by the expert panel as both an ethical requirement and a critical outcome for inclusion in the two COS in inpatient settings: for infants <6 months of age with wasting and/or nutritional oedema and/or underweight (COS 1); and for infants and children 6–59 months of age with severe wasting and/or nutritional oedema (COS 3).

Clinical improvement was also considered a priority outcome for inclusion in these two COS (COS 1 and 3). This outcome should be based on clinical and nutritional signs, requiring clinical judgement. Of note, the outcome clinical improvement emerged from the outcome ‘improvement from severe wasting’, which had been interpreted in two ways by participants: one as clinical improvement, which was seen as critical; the other as improvement based on anthropometry alone, which was not of interest for these COS.

An additional related outcome was oedema resolving, for which consensus was reached about inclusion as a core outcome for the two inpatient setting COS (COS 1 and 3) and for the COS for infants and children 6–59 months of age with severe wasting or nutritional oedema in outpatient/community settings (COS 4).

Morbidity or recovery from morbidity is another outcome that met the consensus criteria for both COS in infants <6 months of age with wasting and/or nutritional oedema and/or underweight (COS 1 and 2) and for the COS on infants and children 6–59 months of age with severe wasting and/or nutritional oedema in inpatient settings (COS 3). The expert panel agreed that this outcome should be based on clinical judgement such as around the resolution of medical problems that prompted admission.

Morbidity was also included as an outcome in the COS for prevention of wasting and nutritional oedema (COS 6). The expert panel highlighted that morbidity data in prevention trials can often be of low quality, without the opportunity to assess morbidity directly in children. They, therefore, agreed that objective information, such as inpatient admissions and/or outpatient clinic visits, should be used whenever possible.

#### Breastfeeding indicators and weight change for infants <6 months

The expert panel agreed that breastfeeding indicators be included for both COS focusing on infants <6 months of age with wasting and/or nutritional oedema and/or underweight (COS 1 and 2). Exclusive breastfeeding of infants <6 months of age is one potential indicator that could be used in trials. The expert panel noted that effective breastfeeding may be considered alongside other outcomes included in the COS, such as weight change.

#### Anthropometric outcomes differing by population and setting

MUAC was included in all COS in outpatient/community settings (COS 2, 4 and 5) and in the COS for prevention of wasting and nutritional oedema (COS 6). The expert panel agreed on excluding WLZ or WHZ from all the COS. Here it was emphasised that these outcomes are distinct from definitions or identification criteria for wasting, with due acknowledgement of the importance of WLZ or WHZ for identifying wasting (along with MUAC for infants and children above 6 months of age only) recommended by WHO. They did not feel that there was added value in including WLZ or WHZ in the COS on management of wasting (COS 1–5). However, it was included in the COS for prevention of wasting and nutritional oedema (COS 6), especially due to its importance in accurately assessing incidence and prevalence of wasting.

The expert panel also came to agreement on excluding anthropometric outcomes from the COS for interventions in inpatient settings in infants and children 6–59 months of age with severe wasting and/or nutritional oedema (COS 3). They agreed that there was more value in other outcomes in this population in inpatient settings, particularly mortality and clinical outcomes, and that interventions in inpatient settings typically do and should aim to impact these outcomes. However, weight change (g/kg/day) was included in the COS on infants <6 months of age with wasting and/or nutritional oedema and/or underweight in inpatient settings (COS 1). The expert panel felt that this was an important outcome specific to this age group even in inpatient settings because of the importance of these infants gaining weight as a potential indicator of treatment response and success.

Weight change (g/kg/day) was also included in the three COS in outpatient/community settings (COS 2, 4 and 5). The expert panel noted, however, that the optimal rate of weight gain in all these populations remains unknown and requires further research.

#### Sustained recovery in trials with postexit anthropometric outcome data

Sustained recovery met consensus criteria for all five COS on management of infants and children with wasting and nutritional oedema (COS 1–5). The expert panel felt that it is a critical outcome that encompasses other outcomes, which were not included in the COS, such as relapse and readmission.

For the two inpatient COS including infants <6 months of age with wasting and/or nutritional oedema and/or underweight (COS 1) and infants and children 6–59 months of age with severe wasting and/or nutritional oedema (COS 3), participants suggested that sustained recovery should be examined in any trial with postexit anthropometric outcome data.

The expert panel noted that for all the COS in inpatient or outpatient/community settings, sustained recovery would ideally be defined as anthropometric recovery sustained for at least 6 months. They acknowledged that the follow-up duration may be dependent on the type of intervention being examined. For instance, a longer duration of follow-up may be appropriate for interventions that aim to impact the home and care environment or that have a specific objective of preventing relapse. On the other hand, follow-up duration may be shorter for interventions that are related to direct protocols for management of wasting.

Other related outcomes that were prioritised included anthropometric recovery and non-response for the three outpatient/community COS (COS 2, 4 and 5); time to anthropometric recovery for the COS on infants and children 6–59 months of age with severe wasting or nutritional oedema in outpatient/community settings (COS 4) and deterioration to severe wasting and/or nutritional oedema for the COS on infants and children 6–59 months of age with moderate wasting in outpatient/community settings (COS 5). The expert panel felt that having outcomes that include a time frame, including non-response and time to anthropometric recovery, would be important especially for evaluations of resources and cost-effectiveness.

#### Prevalence or incidence of wasting dependent on the prevention intervention and trial design

For the COS on prevention of wasting and nutritional oedema (COS 6), both prevalence and incidence met consensus criteria, based on MUAC, WHZ and or nutritional oedema. The expert panel specifically noted the challenges with collecting data on incidence, which can be more resource intensive. The expert panel agreed that prevalence should be reported in population-based or community-based trials; incidence should be reported in individually randomised trials, and in population-based or community-based trials whenever possible. This applies to prevalence and incidence of wasting and/or nutritional oedema as well as prevalence and incidence of severe wasting and/or nutritional oedema specifically.

The expert panel made note of the importance of seasonality for prevalence data particularly. Researchers should ensure that they consider the impacts of seasonality and if relevant, take this into account by examining prevalence at baseline and follow-up at the same time of year, for instance. This may also enable generating useful cost-effectiveness information in addition to effectiveness data.

## Discussion

The development of COS for effectiveness trials on prevention and management of wasting and nutritional oedema emerged as a priority following the 2023 WHO guideline development process. To the best of our knowledge, this is the first time that COS have been developed for infants and children with any form of malnutrition, and the COS for prevention of wasting and nutritional oedema is one of few COS specifically focused on prevention.

These COS serve an important purpose in outlining the minimum set of critical outcomes that should be measured and reported on in effectiveness trials in infants and children with wasting and nutritional oedema. Establishing COS for effectiveness trials on wasting and nutritional oedema will enable researchers to standardise reporting and analyses of outcome data. This will make it easier for results of multiple trials to be compared, contrasted and combined when appropriate. It will also enable future GDGs to make decisions around critical outcomes for guideline questions of interest on wasting and nutritional oedema.

We approached the development of these COS from a child health perspective with children’s health, growth and development at the forefront, aligning with the 2023 WHO guideline on wasting and nutritional oedema,^1^ as opposed to a broader public health perspective, for example. While there has been an increase in the number of COS on child health over time, there have been few of high quality in this area.^21^ These COS on wasting and nutritional oedema fill an important gap in the field of child health and set the stage for future high-quality child health COS.

Furthermore, there have been limited COS established specifically with guideline development in mind.^22^ While potentially very useful and recommended,^23^ it is currently rare for COS to be routinely used during guideline development to select the most critical outcomes for decision-making to develop recommendations.^24^ These COS for wasting and nutritional oedema are intended to bridge the gap from research to recommendations. The COS will enable pooling of data from different trials in meta-analysis, allowing for GRADE assessments for each outcome across multiple studies, and judgements by GDGs to inform recommendations. COS can also reduce risk of bias of studies due to missing outcome data, for instance, which can mean lower overall risk of bias and potentially higher certainty of evidence based on GRADE.^6 25^ These COS should lead to more efficiency in prioritising outcomes for specific guideline questions in the early stages of guideline development—which does not often undergo this same rigorous consensus process—and are a foundation for prioritising outcomes that are most critical for decision-making.

Of note, these COS do not specify the primary outcome for every trial, nor the full set of outcomes that may need to be measured depending on the nature of the intervention being evaluated. Trialists will need to carefully consider their choice of primary outcomes and the full set of outcomes for each trial, in addition to what is included in the relevant COS. Importantly, trials may not be powered to detect differences for all outcomes within the COS, and researchers may cite the COS paper and note this limitation.

Through this COS development process, the expert panel decided that it would not be appropriate to define all outcomes and be too prescriptive in the COS because of the risk of this changing practice. They opted to be more flexible for outcomes that are subjective. This includes those involving judgement, such as through clinical examination or for certain breastfeeding indicators like effective breastfeeding. Though we acknowledge this may increase variability across trials, we will aim to understand this further as these COS are applied. The expert panel also highlighted that an aim of the COS would be to agree on the critical outcomes and to guide researchers in how to optimally report data, but not how to interpret data. There also needs to be further consideration of outcomes for research studies versus as indicators for programmes on wasting and nutritional oedema, which is beyond the scope of this COS development exercise.

With regards to specific outcomes, the expert panel acknowledged that there needs to be further exploration beyond what was possible through this process to better understand outcomes and how they relate to each other, potentially supported by the development of a conceptual framework of surrogate and target outcomes. Specific outcomes require more research, such as the optimal rate of weight gain, which remains unknown. Non-response is another outcome that requires more consideration and conceptualisation, as well as an understanding of reasons for non-response, such as illness and/or disability in order to help address this problem in practice. The expert panel also noted that additional indicators and outcomes along the impact pathway should be examined particularly for prevention trials, to consider how interventions may impact incidence or prevalence of wasting and nutritional oedema as endpoints.

A 2021 systematic review about COS uptake showed that uptake varies greatly across different areas of health, but that there had been no COS uptake studies for child health.^26^ In studies that have been published on COS uptake, the main reason for low uptake was lack of awareness of the existence of COS.^27^ To this effect, we plan to disseminate that these COS as widely as possible to partners and collaborators such as UN agencies, researchers and funders. We will aim to collect information on uptake of these COS across research settings and within systematic reviews and to solicit feedback from those applying the COS to further understand their utility and areas for future development.

### Limitations

One limitation of this COS development process is that patients and caregivers were not included in the development. There are concerns around the sensitivity of discussing outcomes like mortality in infants and children, which would make it challenging to include their caregivers or families. We also acknowledge that the two survey rounds and consensus meetings were all virtual, which could have made for challenges in identifying and involving caregivers of infants and children with wasting and nutritional oedema across settings and contexts. We unfortunately did not have the funding or capacity for participatory work with in-person meetings or interviews translated into relevant languages.

We do feel that involving caregivers would be an important next step in the evolution of these COS to ensure that outcomes are truly considered important by patients, caregivers and communities based on their lived experiences. This issue was highlighted in the aforementioned systematic review about COS uptake, emphasising a lack of patient involvement and the need to better understand what is relevant to patients.^26^

That said, we commissioned a qualitative evidence synthesis for the WHO guideline on wasting and nutritional oedema specifically focused on values and preferences as they relate to the outcomes prioritised for the guideline questions. Based on the available qualitative evidence in different contexts, the prioritised outcomes were seen by families and health workers to be important.^27^ The judgement by the GDG for the 2023 WHO guideline across the guideline questions was that there would probably be no important uncertainty or variability around how much people value the prioritised outcomes across settings.

## Conclusion

We developed six COS for effectiveness trials on the prevention and management of wasting and nutritional oedema, in inpatient and outpatient/community settings. Uptake of these COS will streamline and strengthen primary research, future guidelines and related decision-making, including informing recommendations when the 2023 WHO guideline on the prevention and management of wasting and nutritional oedema is updated based on the best available evidence from effectiveness trials across contexts.

## Supplementary material

10.1136/bmjgh-2024-017225 online supplemental file 1

## Data Availability

All data relevant to the study are included in the article or uploaded as supplementary information.
