# Supplementary material for: Development of core outcome sets for effectiveness trials focused on infants and children with wasting and nutritional oedema
Source: BMJ Glob Health. 2025 Oct 2;10(Suppl 5):e017225. doi: 10.1136/bmjgh-2024-017225 (PMC12496088; doi:10.1136/bmjgh-2024-017225)
Supplement: online supplemental file 1 [file bmjgh-10-Suppl_5-s001.docx]

**Development of core outcome sets for effectiveness trials focused on infants and children with wasting and nutritional oedema**

***Supplemental file***

Allison I Daniel, Kirrily de Polnay, Jaden Bendabenda, Zita Weise Prinzo, Michael McCaul, Celeste E Naude

**Supplemental Table 1. Number of outcomes meeting criteria for consensus after round 2 of the survey and after consensus meetings for each of the six core outcome sets.**

| **Core outcome set** | **Number of outcomes meeting criteria for consensus after round 2 of the survey** | **Number of outcomes included in the final COS after consensus meetings** |
| --- | --- | --- |
| 1) Infants less than 6 months of age with wasting and/or nutritional oedema and/or underweight – *inpatient settings* | 15 | 8 |
| 2) Infants less than 6 months of age with wasting and/or nutritional oedema and/or underweight – *outpatient/community settings* | 15 | 9 |
| 3) Infants and children 6-59 months of age with severe wasting and/or nutritional oedema – *inpatient settings* | 16 | 6 |
| 4) Infants and children 6-59 months of age with severe wasting or nutritional oedema – *outpatient/community settings* | 15 | 9 |
| 5) Infants and children 6-59 months of age with moderate wasting – *outpatient/community settings* | 12 | 8 |
| 6) Prevention of wasting and nutritional oedema | 11 | 6 |

**Overall results from round 1 survey**

**1) Infants less than 6 months of age with wasting and/or nutritional oedema and/or underweight *– inpatient settings***

Mortality

|  | **Mean (SD)** | **Median (IQR)** |
| --- | --- | --- |
| mortality | 8.4 (1.1) | 9 (8.5, 9.0) |

Anthropometric outcomes

|  | **Mean (SD)** | **Median (IQR)** |
| --- | --- | --- |
| WLZ | 6.0 (2.8) | 7 (4.5, 8.5) |
| weight | 7.2 (1.9) | 8 (6.0, 9.0) |
| MUAC | 6.0 (2.8) | 7 (4.0, 8.0) |
| WAZ | 6.9 (2.6) | 8 (6.5, 9.0) |
| LAZ | 4.7 (2.6) | 5 (2.5, 6.0) |
| length | 4.5 (2.8) | 4 (1.5, 7.0) |

Breastfeeding outcomes

|  | **Mean (SD)** | **Median (IQR)** |
| --- | --- | --- |
| breastfeeding indicators | 7.8 (1.8) | 9 (6.8, 9.0) |
| breastmilk intake | 5.7 (2.4) | 5.5 (4.0, 7.3) |

Morbidity

|  | **Mean (SD)** | **Median (IQR)** |
| --- | --- | --- |
| morbidity or recovery from morbidity | 7.6 (1.4) | 8 (7.0, 9.0) |
| duration of diarrhea | 6.0 (1.7) | 6 (5.0, 7.0) |

Hospital stay

|  | **Mean (SD)** | **Median (IQR)** |
| --- | --- | --- |
| duration of hospital stay | 6.6 (2.0) | 7 (5.8, 8.0) |

Adverse events

|  | **Mean (SD)** | **Median (IQR)** |
| --- | --- | --- |
| hospital acquired infections | 6.9 (1.7) | 7 (6.0, 8.3) |
| clinical deterioration | 7.2 (1.9) | 7.5 (7.0, 8.3) |
| duration and intensity of osmotic diarrhea | 5.7 (1.8) | 5 (5.0, 7.0) |
| not tolerating feeds | 6.5 (1.9) | 7 (5.0, 8.0) |

Care pathway outcomes

|  | **Mean (SD)** | **Median (IQR)** |
| --- | --- | --- |
| anthropometric recovery | 6.9 (2.5) | 8 (6.8, 8.0) |
| improvement from severe wasting | 6.3 (2.4) | 7 (5.5, 8.0) |
| time to recovery | 6.1 (2.2) | 6 (5.0, 8.0) |
| non-response | 7.4 (1.7) | 8 (7.0, 8.3) |
| relapse | 7.6 (1.4) | 8 (7.0, 8.5) |
| relapse to moderate wasting | 5.3 (2.6) | 6 (3.0, 7.0) |
| relapse to severe wasting | 6.1 (2.7) | 7 (5.5, 8.0) |
| readmission | 7.6 (1.0) | 8 (7.0, 8.0) |
| sustained recovery | 7.2 (1.9) | 8 (6.5, 8.5) |

Child development

|  | **Mean (SD)** | **Median (IQR)** |
| --- | --- | --- |
| child development | 5.0 (2.2) | 5.5 (4.0, 7.0) |

**2) Infants less than 6 months of age with wasting and/or nutritional oedema and/or underweight *– outpatient/community settings***

Mortality

|  | **Mean (SD)** | **Median (IQR)** |
| --- | --- | --- |
| mortality | 8.0 (1.7) | 9 (7.5, 9.0) |

Anthropometric outcomes

|  | **Mean (SD)** | **Median (IQR)** |
| --- | --- | --- |
| WLZ | 6.3 (2.8) | 7 (5.0, 9.0) |
| weight | 7.5 (1.9) | 8 (7.0, 9.0) |
| MUAC | 7.0 (2.1) | 8 (6.0, 9.0) |
| WAZ | 7.5 (2.3) | 8 (7.8, 9.0) |
| LAZ | 5.2 (2.8) | 5.5 (3.0, 8.0) |
| length | 5.1 (2.7) | 6 (2.8, 7.0) |

Breastfeeding outcomes

|  | **Mean (SD)** | **Median (IQR)** |
| --- | --- | --- |
| breastfeeding indicators | 7.7 (1.7) | 8 (7.0, 9.0) |
| breastmilk intake | 5.2 (2.9) | 5 (2.8, 8.0) |

Morbidity

|  | **Mean (SD)** | **Median (IQR)** |
| --- | --- | --- |
| morbidity or recovery from morbidity | 7.1 (2.2) | 8 (7.0, 9.0) |

Care pathway outcomes

|  | **Mean (SD)** | **Median (IQR)** |
| --- | --- | --- |
| anthropometric recovery | 8.0 (1.5) | 8 (8.0, 9.0) |
| improvement from severe wasting | 7.1 (2.0) | 8 (6.8, 8.0) |
| time to recovery | 6.8 (1.9) | 7 (6.0, 8.0) |
| non-response | 7.6 (1.4) | 8 (7.0, 9.0) |
| relapse | 7.7 (1.3) | 8 (7.0, 9.0) |
| relapse to moderate wasting | 5.9 (2.2) | 6 (5.0, 7.3) |
| relapse to severe wasting | 6.6 (2.4) | 7 (6.0, 8.0) |
| readmission | 7.8 (1.3) | 8 (7.0, 9.0) |
| sustained recovery | 7.4 (1.6) | 8 (7.0, 8.5) |

Child development

|  | **Mean (SD)** | **Median (IQR)** |
| --- | --- | --- |
| child development | 6.0 (2.1) | 6 (5.8, 7.3) |

**3) Infants and children 6-59 months of age with severe wasting and/or nutritional oedema *– inpatient settings***

Mortality

|  | **Mean (SD)** | **Median (IQR)** |
| --- | --- | --- |
| mortality | 8.7 (0.8) | 9 (9.0, 9.0) |

Anthropometric outcomes

|  | **Mean (SD)** | **Median (IQR)** |
| --- | --- | --- |
| WHZ | 7.3 (2.6) | 8 (7.0, 9.0) |
| weight | 6.1 (2.8) | 7 (4.0, 9.0) |
| MUAC | 7.2 (2.7) | 8 (7.0, 9.0) |
| WAZ | 6.5 (2.9) | 8 (5.8, 9.0) |
| HAZ | 5.0 (2.7) | 5.5 (7.0, 2.8) |
| height | 5.1 (2.9) | 5 (3.5, 7.0) |

Morbidity

|  | **Mean (SD)** | **Median (IQR)** |
| --- | --- | --- |
| morbidity or recovery from morbidity | 7.7 (1.3) | 8 (7.0, 9.0) |
| duration of diarrhea | 6.0 (1.8) | 6 (5.0, 6.0) |

Hospital stay

|  | **Mean (SD)** | **Median (IQR)** |
| --- | --- | --- |
| duration of hospital stay | 6.9 (1.9) | 7 (6.8, 8.0) |

Adverse events

|  | **Mean (SD)** | **Median (IQR)** |
| --- | --- | --- |
| hospital acquired infections | 7.0 (1.5) | 7 (6.0, 8.0) |
| clinical deterioration | 7.4 (1.3) | 7 (7.0, 8.0) |
| duration and intensity of osmotic diarrhea | 5.4 (1.8) | 6 (5.0, 7.0) |
| not tolerating feeds | 6.3 (2.0) | 7 (5.0, 8.0) |

Clinical outcomes

|  | **Mean (SD)** | **Median (IQR)** |
| --- | --- | --- |
| time to full rehydration | 5.3 (2.5) | 6 (2.8, 7.0) |
| duration of NPO and IV maintenance fluids used | 4.9 (2.4) | 5 (4.0, 7.0) |

Care pathway outcomes

|  | **Mean (SD)** | **Median (IQR)** |
| --- | --- | --- |
| anthropometric recovery | 7.0 (2.4) | 8 (6.8, 8.3) |
| improvement from severe wasting | 6.7 (2.4) | 8 (8.0, 6.0) |
| time to recovery | 6.5 (2.1) | 7 (6.0, 7.3) |
| non-response | 7.1 (2.2) | 8 (6.8, 8.3) |
| relapse | 7.4 (1.8) | 8 (7.0, 8.0) |
| relapse to moderate wasting | 5.4 (2.5) | 6 (4.8, 7.0) |
| relapse to severe wasting | 7.0 (1.9) | 7 (6.8, 8.0) |
| readmission | 7.7 (1.2) | 8 (8.3, 7.0) |
| sustained recovery | 7.3 (2.0) | 8 (7.0, 9.0) |

Child development

|  | **Mean (SD)** | **Median (IQR)** |
| --- | --- | --- |
| child development | 5.6 (2.3) | 6 (4.0, 7.3) |

**4) Infants and children 6-59 months of age with severe wasting or nutritional oedema –*outpatient/community settings***

Mortality

|  | **Mean (SD)** | **Median (IQR)** |
| --- | --- | --- |
| mortality | 8.0 (1.9) | 9 (8.0, 9.0) |

Anthropometric outcomes

|  | **Mean (SD)** | **Median (IQR)** |
| --- | --- | --- |
| WHZ | 7.8 (2.0) | 9 (7.0, 9.0) |
| weight | 7.5 (2.0) | 8 (6.0, 9.0) |
| MUAC | 8.2 (1.1) | 9 (8.0, 9.0) |
| WAZ | 7.3 (2.4) | 8 (7.0, 9.0) |
| HAZ | 5.8 (2.7) | 6.5 (4.0, 8.0) |
| height | 5.9 (2.5) | 6 (4.0, 8.0) |

Care pathway outcomes

|  | **Mean (SD)** | **Median (IQR)** |
| --- | --- | --- |
| anthropometric recovery | 7.9 (1.6) | 8 (8.0, 9.0) |
| improvement from severe wasting | 7.6 (1.7) | 8 (7.0, 8.0) |
| time to recovery | 7.2 (1.9) | 7 (6.0, 9.0) |
| non-response | 7.5 (1.4) | 8 (7.0, 8.0) |
| relapse | 7.6 (1.4) | 8 (7.0, 9.0) |
| relapse to moderate wasting | 5.8 (2.4) | 6 (5.0, 8.0) |
| relapse to severe wasting | 6.6 (2.4) | 7 (6.0, 8.0) |
| readmission | 7.5 (1.6) | 8 (7.0, 8.3) |
| sustained recovery | 7.4 (1.7) | 8 (7.0, 8.0) |

Child development

|  | **Mean (SD)** | **Median (IQR)** |
| --- | --- | --- |
| child development | 6.6 (2.0) | 7 (5.0, 8.0) |

**5) Infants and children 6-59 months of age with moderate wasting – *outpatient/community settings***

Mortality

|  | **Mean (SD)** | **Median (IQR)** |
| --- | --- | --- |
| mortality | 7.3 (2.5) | 8.5 (6.8, 9.0) |

Anthropometric outcomes

|  | **Mean (SD)** | **Median (IQR)** |
| --- | --- | --- |
| WHZ | 7.8 (1.6) | 8 (7.0, 9.0) |
| weight | 7.0 (2.5) | 8 (6.0, 9.0) |
| MUAC | 8.3 (1.1) | 9 (8.0, 9.0) |
| WAZ | 7.2 (2.4) | 8 (7.0, 9.0) |
| HAZ | 6.0 (2.7) | 7 (4.0, 8.0) |
| height | 5.8 (2.8) | 6.5 (4.0, 9.0) |

Care pathway outcomes

|  | **Mean (SD)** | **Median (IQR)** |
| --- | --- | --- |
| anthropometric recovery | 8.0 (1.4) | 8 (8.0, 9.0) |
| time to recovery | 7.0 (1.9) | 7 (6.0, 8.0) |
| non-response | 7.4 (1.8) | 8 (7.0, 9.0) |
| deterioration to severe wasting | 7.6 (1.7) | 8 (8.0, 8.0) |
| relapse | 7.3 (1.6) | 8 (7.0, 8.0) |
| relapse to moderate wasting | 6.3 (1.9) | 6 (5.0, 8.0) |
| relapse to severe wasting | 6.7 (2.5) | 7 (6.0, 8.0) |
| readmission | 7.3 (1.8) | 8 (7.0, 8.3) |
| sustained recovery | 7.2 (2.1) | 8 (7.0, 9.0) |

Child development

|  | **Mean (SD)** | **Median (IQR)** |
| --- | --- | --- |
| child development | 6.5 (2.0) | 7 (5.0, 8.0) |

**6) Prevention of wasting and nutritional oedema**

Mortality

|  | **Mean (SD)** | **Median (IQR)** |
| --- | --- | --- |
| mortality | 6.3 (2.8) | 7 (4.8, 9.0) |

Incidence and prevalence

|  | **Mean (SD)** | **Median (IQR)** |
| --- | --- | --- |
| prevalence of wasting | 7.2 (1.9) | 8 (7.0, 9.0) |
| prevalence of moderate wasting | 6.9 (1.9) | 7 (6.0, 9.0) |
| prevalence of severe wasting | 7.2 (2.0) | 8 (6.0, 8.0) |
| incidence of wasting | 7.6 (1.0) | 8 (7.0, 8.0) |
| incidence of severe wasting | 7.6 (1.6) | 8 (7.0, 9.0) |
| cumulative incidence of wasting | 7.5 (1.2) | 7 (7.0, 8.5) |
| cumulative incidence of severe wasting | 7.3 (1.9) | 8 (6.0, 9.0) |
| deterioration to severe wasting | 7.4 (1.2) | 7.5 (6.8, 8.0) |
| prevalence of underweight | 6.7 (1.9) | 7 (6.0, 8.0) |

Anthropometric outcomes

|  | **Mean (SD)** | **Median (IQR)** |
| --- | --- | --- |
| WHZ | 7.0 (2.6) | 8 (6.0, 9.0) |
| MUAC | 7.8 (2.2) | 9 (8.0, 9.0) |
| WAZ | 7.1 (2.5) | 8 (6.8, 9.0) |

Morbidity

|  | **Mean (SD)** | **Median (IQR)** |
| --- | --- | --- |
| morbidity | 6.8 (2.0) | 7 (6.5, 8.0) |
| prevalence of diarrhea | 5.3 (2.6) | 6 (4.0, 7.0) |
| incidence of diarrhea | 6.2 (1.9) | 7 (5.0, 8.0) |
| prevalence of cough or respiratory infection | 5.2 (2.5) | 6 (3.0, 7.0) |
| incidence of cough or respiratory infection | 5.9 (2.0) | 6 (4.8, 8.0) |
| prevalence of fever | 4.8 (2.5) | 5 (2.8, 7.0) |
| incidence of fever | 5.0 (2.5) | 5 (3.0, 7.0) |

**Overall results from round 2 survey**

**1) Infants less than 6 months of age with wasting and/or nutritional oedema and/or underweight *– inpatient settings***

Mortality

|  | **Mean (SD)** | **Median (IQR)** | **Consensus** |
| --- | --- | --- | --- |
| mortality | 8.4 (1.4) | 9 (9.0, 9.0) | Yes |

Anthropometric outcomes

|  | **Mean (SD)** | **Median (IQR)** | **Consensus** |
| --- | --- | --- | --- |
| WLZ | 6.0 (2.4) | 6 (5.0, 8.0) | No |
| weight | 7.6 (1.8) | 8 (7.0, 9.0) | Yes |
| MUAC | 6.4 (2.2) | 7 (5.0, 8.0) | No |
| WAZ | 7.5 (1.6) | 8 (7.0, 8.0) | Yes |
| LAZ | 5.1 (2.2) | 5 (3.8, 7.0) | No |
| length | 5.0 (2.4) | 5 (4.0, 7.0) | No |
| body composition | 5.4 (1.8) | 5.5 (4.8, 6.3) | No |

Oedema resolving

|  | **Mean (SD)** | **Median (IQR)** | **Consensus** |
| --- | --- | --- | --- |
| oedema resolving | 7.1 (1.6) | 7 (6.0, 8.0) | No (69.57%) |

Breastfeeding outcomes

|  | **Mean (SD)** | **Median (IQR)** | **Consensus** |
| --- | --- | --- | --- |
| breastfeeding indicators | 7.7 (2.0) | 9 (7.0, 9.0) | Yes |
| breastmilk intake | 5.7 (2.1) | 6 (5.0, 7.0) | No |

Morbidity

|  | **Mean (SD)** | **Median (IQR)** | **Consensus** |
| --- | --- | --- | --- |
| morbidity or recovery from morbidity | 7.8 (1.0) | 8 (7.0, 8.0) | Yes |
| duration of diarrhea | 5.9 (1.6) | 6 (5.0, 7.0) | No |

Hospital stay

|  | **Mean (SD)** | **Median (IQR)** | **Consensus** |
| --- | --- | --- | --- |
| duration of hospital stay | 6.3 (1.8) | 7 (6.0, 7.0) | No |

Adverse events

|  | **Mean (SD)** | **Median (IQR)** | **Consensus** |
| --- | --- | --- | --- |
| hospital acquired infections | 6.2 (1.3) | 6 (6.0, 7.0) | No |
| clinical deterioration | 7.2 (1.4) | 7 (7.0, 8.0) | Yes |
| duration and intensity of osmotic diarrhea | 5.6 (1.5) | 6 (5.0, 7.0) | No |
| not tolerating feeds | 6.6 (1.6) | 7 (6.0, 7.0) | Yes |

Care pathway outcomes

|  | **Mean (SD)** | **Median (IQR)** | **Consensus** |
| --- | --- | --- | --- |
| anthropometric recovery | 7.0 (2.1) | 8 (7.0, 8.0) | Yes |
| improvement from severe wasting | 6.9 (1.9) | 7 (6.0, 8.0) | Yes |
| time to recovery | 6.5 (1.9) | 6 (6.0, 8.0) | No |
| non-response | 7.2 (1.5) | 8 (7.0, 8.0) | Yes |
| relapse | 7.2 (1.6) | 8 (7.0, 8.0) | Yes |
| relapse to moderate wasting | 5.6 (1.5) | 6 (5.0, 7.0) | No |
| relapse to severe wasting | 6.3 (1.7) | 6.5 (6.0, 7.0) | No |
| readmission | 7.1 (1.8) | 8 (7.0, 8.0) | Yes |
| sustained recovery | 7.2 (1.5) | 8 (7.0, 8.0) | Yes |
| loss to follow-up or default | 6.3 (2.2) | 7 (5.8, 7.0) | No |
| successful transfer to outpatient care | 6.6 (2.0) | 7 (6.0, 8.0) | Yes |

Child development

|  | **Mean (SD)** | **Median (IQR)** | **Consensus** |
| --- | --- | --- | --- |
| child development | 5.6 (1.9) | 6 (5.0, 7.0) | No |
| other functional outcomes (ability to do “x”) | 5.2 (2.0) | 5 (4.0, 7.0) | No |

Biomarkers

|  | **Mean (SD)** | **Median (IQR)** | **Consensus** |
| --- | --- | --- | --- |
| immune function biomarkers | 5.3 (2.3) | 6 (3.3, 7.0) | No |
| gut inflammation biomarkers | 5.4 (2.4) | 6 (3.5, 7.0) | No |

**2) Infants less than 6 months of age with wasting and/or nutritional oedema and/or underweight *– outpatient/community settings***

Mortality

|  | **Mean (SD)** | **Median (IQR)** | **Consensus** |
| --- | --- | --- | --- |
| mortality | 8.1 (1.5) | 9 (8.0, 9.0) | Yes |

Anthropometric outcomes

|  | **Mean (SD)** | **Median (IQR)** | **Consensus** |
| --- | --- | --- | --- |
| WLZ | 6.7 (2.2) | 7 (6.0, 8.0) | No |
| weight | 7.7 (2.0) | 8 (8.0, 9.0) | Yes |
| MUAC | 7.6 (1.7) | 8 (6.0, 9.0) | Yes |
| WAZ | 7.8 (1.5) | 8 (7.8, 9.0) | Yes |
| LAZ | 5.5 (2.1) | 5.5 (5.0, 6.3) | No |
| length | 5.4 (2.3) | 6 (4.0, 7.0) | No |
| body composition | 5.3 (1.8) | 5 (4.8, 7.0) | No |

Oedema resolving

|  | **Mean (SD)** | **Median (IQR)** | **Consensus** |
| --- | --- | --- | --- |
| oedema resolving | 7.3 (1.6) | 7 (7.0, 8.0) | Yes |

Breastfeeding outcomes

|  | **Mean (SD)** | **Median (IQR)** | **Consensus** |
| --- | --- | --- | --- |
| breastfeeding indicators | 7.8 (1.9) | 8 (7.0, 9.0) | Yes |
| breastmilk intake | 5.8 (2.2) | 6 (5.0, 7.0) | No |

Morbidity

|  | **Mean (SD)** | **Median (IQR)** | **Consensus** |
| --- | --- | --- | --- |
| morbidity or recovery from morbidity | 7.2 (1.8) | 8 (7.0, 8.0) | Yes |

Care pathway outcomes

|  | **Mean (SD)** | **Median (IQR)** | **Consensus** |
| --- | --- | --- | --- |
| anthropometric recovery | 7.8 (1.6) | 8 (8.0, 9.0) | Yes |
| improvement from severe wasting | 7.2 (1.7) | 8 (7.0, 8.0) | Yes |
| time to recovery | 6.6 (2.1) | 7 (6.0, 8.0) | No |
| non-response | 7.6 (1.1) | 8 (7.0, 8.0) | Yes |
| relapse | 7.5 (1.6) | 8 (7.0, 9.0) | Yes |
| relapse to moderate wasting | 6.1 (1.9) | 6 (5.0, 7.0) | No |
| relapse to severe wasting | 7.1 (1.3) | 7 (7.0, 8.0) | Yes |
| readmission | 7.7 (1.4) | 8 (7.0, 9.0) | Yes |
| sustained recovery | 7.3 (1.5) | 8 (7.0, 8.0) | Yes |
| duration of treatment in care pathway | 6.4 (1.6) | 7 (5.8, 7.0) | No |
| loss to follow-up or default | 6.9 (1.8) | 7 (7.0, 8.0) | Yes |

Child development

|  | **Mean (SD)** | **Median (IQR)** | **Consensus** |
| --- | --- | --- | --- |
| child development | 5.9 (2.1) | 6 (5.0, 7.0) | No |
| other functional outcomes (ability to do “x”) | 5.4 (2.1) | 6 (4.0, 7.0) | No |

Biomarkers

|  | **Mean (SD)** | **Median (IQR)** | **Consensus** |
| --- | --- | --- | --- |
| immune function biomarkers | 4.9 (2.2) | 5 (3.0, 7.0) | No |
| gut inflammation biomarkers | 5.0 (2.1) | 5 (3.3, 7.0) | No |

**3) Infants and children 6-59 months of age with severe wasting and/or nutritional oedema *– inpatient settings***

Mortality

|  | **Mean (SD)** | **Median (IQR)** | **Consensus** |
| --- | --- | --- | --- |
| mortality | 8.4 (1.7) | 9 (9.0, 9.0) | Yes |

Anthropometric outcomes

|  | **Mean (SD)** | **Median (IQR)** | **Consensus** |
| --- | --- | --- | --- |
| WHZ | 7.3 (2.5) | 8 (8.0, 9.0) | Yes |
| weight | 7.0 (2.1) | 7 (7.0, 8.0) | Yes |
| MUAC | 7.9 (2.1) | 8 (8.0, 9.0) | Yes |
| WAZ | 7.0 (2.4) | 8 (7.0, 8.0) | Yes |
| HAZ | 5.5 (2.3) | 6 (4.0, 7.0) | No |
| height | 5.6 (2.3) | 6 (4.8, 7.0) | No |
| body composition | 5.8 (1.9) | 6 (5.0, 7.0) | No |

Oedema resolving

|  | **Mean (SD)** | **Median (IQR)** | **Consensus** |
| --- | --- | --- | --- |
| oedema resolving | 7.6 (1.0) | 7 (7.0, 8.5) | Yes |

Morbidity

|  | **Mean (SD)** | **Median (IQR)** | **Consensus** |
| --- | --- | --- | --- |
| morbidity or recovery from morbidity | 7.7 (1.0) | 8 (7.0, 8.0) | Yes |
| duration of diarrhea | 6.0 (1.6) | 6 (5.8, 7.0) | No |

Hospital stay

|  | **Mean (SD)** | **Median (IQR)** | **Consensus** |
| --- | --- | --- | --- |
| duration of hospital stay | 6.9 (1.8) | 7 (7.0, 8.0) | Yes |

Adverse events

|  | **Mean (SD)** | **Median (IQR)** | **Consensus** |
| --- | --- | --- | --- |
| hospital acquired infections | 6.4 (1.6) | 7 (6.0, 7.0) | No |
| clinical deterioration | 7.1 (1.6) | 7 (7.0, 8.0) | Yes |
| duration and intensity of osmotic diarrhea | 5.7 (1.5) | 6 (5.0, 7.0) | No |
| not tolerating feeds | 6.4 (1.5) | 7 (6.0, 7.0) | No |

Clinical outcomes

|  | **Mean (SD)** | **Median (IQR)** | **Consensus** |
| --- | --- | --- | --- |
| time to full rehydration | 5.8 (1.7) | 6 (6.0, 7.0) | No |
| duration of NPO and IV maintenance fluids used | 5.5 (1.6) | 5.5 (5.0, 6.8) | No |

Appetite

|  | **Mean (SD)** | **Median (IQR)** | **Consensus** |
| --- | --- | --- | --- |
| Appetite | 6.5 (1.9) | 7 (6.0, 8.0) | No |

Care pathway outcomes

|  | **Mean (SD)** | **Median (IQR)** | **Consensus** |
| --- | --- | --- | --- |
| anthropometric recovery | 7.4 (2.0) | 8 (7.0, 8.0) | Yes |
| improvement from severe wasting | 7.1 (2.0) | 8 (6.0, 8.0) | Yes |
| time to recovery | 6.8 (1.8) | 7 (7.0, 7.0) | Yes |
| non-response | 7.4 (1.8) | 8 (7.0, 8.0) | Yes |
| relapse | 7.1 (2.3) | 8 (7.0, 8.0) | Yes |
| relapse to moderate wasting | 5.7 (1.9) | 6 (5.0, 6.0) | No |
| relapse to severe wasting | 6.7 (1.8) | 7 (6.0, 8.0) | No |
| readmission | 7.6 (1.5) | 8 (7.0, 8.0) | Yes |
| sustained recovery | 7.4 (1.5) | 8 (7.0, 8.0) | Yes |
| loss to follow-up or default | 6.5 (2.2) | 7 (6.0, 8.0) | No |

Breastfeeding outcomes (up to at least 2 years)

|  | **Mean (SD)** | **Median (IQR)** | **Consensus** |
| --- | --- | --- | --- |
| breastfeeding indicators (up to at least 2 years) | 6.4 (2.4) | 7 (5.0, 8.0) | No |

Child development

|  | **Mean (SD)** | **Median (IQR)** | **Consensus** |
| --- | --- | --- | --- |
| child development | 6.0 (1.8) | 6 (5.0, 7.0) | No |
| other functional outcomes (ability to do “x”) | 5.3 (1.7) | 6 (4.5, 6.5) | No |

Biomarkers

|  | **Mean (SD)** | **Median (IQR)** | **Consensus** |
| --- | --- | --- | --- |
| immune function biomarkers | 5.4 (2.2) | 5.5 (4.3, 7.0) | No |
| gut inflammation biomarkers | 5.3 (2.2) | 6 (4.5, 7.0) | No |

**4) Infants and children 6-59 months of age with severe wasting or nutritional oedema –*outpatient/community settings***

Mortality

|  | **Mean (SD)** | **Median (IQR)** | **Consensus** |
| --- | --- | --- | --- |
| mortality | 8.2 (1.5) | 9 (8.0, 9.0) | Yes |

Anthropometric outcomes

|  | **Mean (SD)** | **Median (IQR)** | **Consensus** |
| --- | --- | --- | --- |
| WHZ | 7.8 (1.9) | 8 (8.0, 9.0) | Yes |
| weight | 7.8 (1.9) | 8 (8.0, 9.0) | Yes |
| MUAC | 8.7 (0.5) | 9 (8.0, 9.0) | Yes |
| WAZ | 7.4 (2.0) | 8 (7.0, 9.0) | Yes |
| HAZ | 6.6 (2.1) | 6 (5.0, 8.0) | No |
| height | 6.2 (2.1) | 6 (5.0, 8.0) | No |
| body composition | 6.0 (1.9) | 6 (5.0, 7.3) | No |

Oedema resolving

|  | **Mean (SD)** | **Median (IQR)** | **Consensus** |
| --- | --- | --- | --- |
| oedema resolving | 7.3 (1.6) | 7 (7.0, 8.5) | Yes |

Care pathway outcomes

|  | **Mean (SD)** | **Median (IQR)** | **Consensus** |
| --- | --- | --- | --- |
| anthropometric recovery | 7.8 (1.7) | 8 (8.0, 9.0) | Yes |
| improvement from severe wasting | 7.6 (1.7) | 8 (8.0, 8.0) | Yes |
| time to recovery | 7.2 (1.8) | 7 (6.8, 8.3) | Yes |
| non-response | 7.8 (0.9) | 8 (7.0, 8.0) | Yes |
| relapse | 7.7 (1.4) | 8 (7.0, 8.3) | Yes |
| relapse to moderate wasting | 6.2 (1.5) | 6 (6.0, 7.0) | No |
| relapse to severe wasting | 7.3 (1.5) | 7 (7.0, 8.0) | Yes |
| readmission | 7.4 (1.5) | 8 (7.0, 8.0) | Yes |
| sustained recovery | 7.3 (1.6) | 7.5 (7.0, 8.0) | Yes |
| loss to follow-up or default | 6.8 (1.8) | 7 (6.0, 8.0) | Yes |

Breastfeeding outcomes (up to at least 2 years)

|  | **Mean (SD)** | **Median (IQR)** | **Consensus** |
| --- | --- | --- | --- |
| breastfeeding indicators (up to at least 2 years) | 6.7 (2.3) | 7 (5.0, 9.0) | No |

Child development

|  | **Mean (SD)** | **Median (IQR)** | **Consensus** |
| --- | --- | --- | --- |
| child development | 6.3 (2.1) | 6 (5.0, 8.0) | No |
| other functional outcomes (ability to do “x”) | 5.7 (1.9) | 6 (5.0, 7.0) | No |

Biomarkers

|  | **Mean (SD)** | **Median (IQR)** | **Consensus** |
| --- | --- | --- | --- |
| immune function biomarkers | 5.3 (2.3) | 6 (4.0, 7.0) | No |
| gut inflammation biomarkers | 5.2 (2.2) | 6 (4.0, 7.0) | No |

**5) Infants and children 6-59 months of age with moderate wasting*–outpatient/community settings***

Mortality

|  | **Mean (SD)** | **Median (IQR)** | **Consensus** |
| --- | --- | --- | --- |
| mortality | 7.7 (1.9) | 8 (7.0, 9.0) | Yes |

Anthropometric outcomes

|  | **Mean (SD)** | **Median (IQR)** | **Consensus** |
| --- | --- | --- | --- |
| WHZ | 7.9 (1.5) | 8 (7.0, 9.0) | Yes |
| weight | 7.5 (2.2) | 8 (7.0, 9.0) | Yes |
| MUAC | 8.6 (0.6) | 9 (8.0, 9.0) | Yes |
| WAZ | 7.2 (1.9) | 7 (7.0, 9.0) | Yes |
| HAZ | 6.3 (2.0) | 7 (5.0, 7.0) | No |
| height | 6.0 (2.2) | 6 (5.0, 7.3) | No |
| body composition | 6 (1.9) | 6 (5.0, 7.3) | No |

Care pathway outcomes

|  | **Mean (SD)** | **Median (IQR)** | **Consensus** |
| --- | --- | --- | --- |
| anthropometric recovery | 8.0 (1.4) | 8 (8.0, 9.0) | Yes |
| time to recovery | 7.0 (1.8) | 7 (6.0, 8.0) | No |
| non-response | 7.5 (1.3) | 8 (7.0, 8.0) | Yes |
| deterioration to severe wasting | 7.6 (1.3) | 8 (7.0, 8.0) | Yes |
| relapse | 7.3 (1.5) | 8 (7.0, 8.0) | Yes |
| relapse to moderate wasting | 6.3 (1.5) | 6 (5.8, 7.0) | No |
| relapse to severe wasting | 6.1 (1.5) | 7 (7.0, 8.0) | Yes |
| readmission | 7.6 (1.4) | 8 (7.0, 8.0) | Yes |
| sustained recovery | 6.4 (1.8) | 7 (6.0, 7.0) | Yes |

Morbidity

|  | **Mean (SD)** | **Median (IQR)** | **Consensus** |
| --- | --- | --- | --- |
| morbidity or recovery from morbidity | 6.4 (1.8) | 7 (6.0, 7.0) | No |

Breastfeeding outcomes (up to at least 2 years)

|  | **Mean (SD)** | **Median (IQR)** | **Consensus** |
| --- | --- | --- | --- |
| breastfeeding indicators (up to at least 2 years) | 6.4 (2.3) | 7 (5.0, 8.0) | No |

Child development

|  | **Mean (SD)** | **Median (IQR)** | **Consensus** |
| --- | --- | --- | --- |
| child development | 6.3 (1.8) | 6 (5.0, 7.0) | No |
| other functional outcomes (ability to do “x”) | 5.6 (1.9) | 6 (4.5, 7.0) | No |

**6) Prevention of wasting and nutritional oedema**

Mortality

|  | **Mean (SD)** | **Median (IQR)** | **Consensus** |
| --- | --- | --- | --- |
| mortality | 7.2 (1.8) | 8 (6.0, 9.0) | No |

Incidence and prevalence

|  | **Mean (SD)** | **Median (IQR)** | **Consensus** |
| --- | --- | --- | --- |
| prevalence of wasting | 7.4 (1.9) | 8 (7.0, 9.0) | Yes |
| prevalence of moderate wasting | 6.9 (1.9) | 7 (6.0, 9.0) | No |
| prevalence of severe wasting | 7.3 (1.8) | 8 (6.0, 9.0) | Yes |
| incidence of wasting | 7.3 (1.5) | 8 (7.0, 8.0) | Yes |
| incidence of severe wasting | 7.7 (1.4) | 8 (7.0, 9.0) | Yes |
| cumulative incidence of wasting | 7.3 (1.5) | 7 (7.0, 8.3) | Yes |
| cumulative incidence of severe wasting | 7.7 (1.5) | 8 (7.0, 9.0) | Yes |
| deterioration to severe wasting | 7.4 (1.7) | 8 (7.0, 9.0) | Yes |
| prevalence of underweight | 6.7 (2.1) | 7 (6.0, 8.0) | No |
| time to first incident of wasting | 6.0 (1.8) | 7 (5.0, 7.0) | No |

Anthropometric outcomes

|  | **Mean (SD)** | **Median (IQR)** | **Consensus** |
| --- | --- | --- | --- |
| WHZ | 7.4 (2.0) | 8 (7.0, 9.0) | Yes |
| MUAC | 8.2 (1.5) | 9 (8.0, 9.0) | Yes |
| WAZ | 7.4 (2.1) | 8 (7.0, 9.0) | Yes |
| HAZ | 6.2 (2.2) | 7 (5.0, 7.0) | No |
| height or length | 5.7 (2.1) | 6 (5.0, 7.0) | No |

Morbidity

|  | **Mean (SD)** | **Median (IQR)** | **Consensus** |
| --- | --- | --- | --- |
| morbidity | 7.0 (1.6) | 7 (6.0, 8.0) | Yes |
| prevalence of diarrhea | 5.8 (2.2) | 6 (5.0, 7.0) | No |
| incidence of diarrhea | 5.9 (1.7) | 6 (6.0, 7.0) | No |
| prevalence of cough or respiratory infection | 5.6 (2.2) | 6 (5.0, 7.0) | No |
| incidence of cough or respiratory infection | 5.8 (1.6) | 6 (5.0, 7.0) | No |
| prevalence of fever | 5.0 (2.6) | 6 (3.0, 7.0) | No |
| incidence of fever | 5.0 (2.1) | 6 (4.0, 6.0) | No |

Infant and young child feeding practices

|  | **Mean (SD)** | **Median (IQR)** | **Consensus** |
| --- | --- | --- | --- |
| infant and young child feeding indicators | 6.6 (2.2) | 7 (6.0, 8.0) | No |
| breastfeeding indicators | 6.4 (2.2) | 7 (5.0, 8.0) | No |

Food insecurity

|  | **Mean (SD)** | **Median (IQR)** | **Consensus** |
| --- | --- | --- | --- |
| food insecurity | 6.3 (2.0) | 7 (6.0, 8.0) | No |

Water, sanitation and hygiene

|  | **Mean (SD)** | **Median (IQR)** | **Consensus** |
| --- | --- | --- | --- |
| water, sanitation and hygiene indicators | 6.0 (2.0) | 7 (6.0, 8.0) | No |

Child development

|  | **Mean (SD)** | **Median (IQR)** | **Consensus** |
| --- | --- | --- | --- |
| child development | 5.9 (1.8) | 6 (5.0, 7.0) | No |

**Outcomes that were excluded during the consensus meetings**

| **1) Infants less than 6 months of age with wasting and/or nutritional oedema and/or underweight – *inpatient settings*** | **2) Infants less than 6 months of age with wasting and/or nutritional oedema and/or underweight – *outpatient/community settings*** | **3) Infants and children 6-59 months of age with severe wasting and/or nutritional oedema – *inpatient settings*** | **4) Infants and children 6-59 months of age with severe wasting or nutritional oedema – *outpatient/community settings*** | **5) Infants and children 6-59 months of age with moderate wasting – *outpatient/community settings*** | **6) Prevention of wasting and nutritional oedema** |
| --- | --- | --- | --- | --- | --- |
| - WAZ - Not tolerating feeds - Anthropometric recovery - Improvement from severe wasting - Non-response - Relapse - Readmission - Successful transfer to outpatient care | - Oedema resolving - Improvement from severe wasting - Relapse - Relapse to severe wasting - Readmission - Loss to follow-up^1^ or default | - WHZ - Weight change - MUAC - WAZ - Duration of hospital stay - Anthropometric recovery - Improvement from severe wasting - Time to recovery - Non-response - Relapse - Readmission | - WHZ - Improvement from severe wasting - Relapse - Relapse to severe wasting - Readmission - Loss to follow-up^1^ or default | - WHZ - Relapse - Relapse to severe wasting - Readmission | - Cumulative incidence of wasting - Cumulative incidence of severe wasting - Deterioration to severe wasting - WHZ - WAZ |

^1^ note that all trials need to report on loss to follow-up as good practice
